# Role of Demographic Dynamics and Conflict in the Population-Area Relationship for Human Languages

**DOI:** 10.1371/journal.pone.0040137

**Published:** 2012-07-18

**Authors:** Susanna C. Manrubia, Jacob B. Axelsen, Damián H. Zanette

**Affiliations:** 1 Centro de Astrobiologa, Instituto Nacional de Técnica Aeroespacial and Consejo Superior de Investigaciones Científicas, Torrejón de Ardoz, Madrid, Spain; 2 Consejo Nacional de Investigaciones Cientficas y Técnicas, Centro Atómico Bariloche and Instituto Balseiro, San Carlos de Bariloche, Argentina; University of Zaragoza, Spain

## Abstract

Many patterns displayed by the distribution of human linguistic groups are similar to the ecological organization described for biological species. It remains a challenge to identify simple and meaningful processes that describe these patterns. The population size distribution of human linguistic groups, for example, is well fitted by a log-normal distribution that may arise from stochastic demographic processes. As we show in this contribution, the distribution of the area size of home ranges of those groups also agrees with a log-normal function. Further, size and area are significantly correlated: the number of speakers 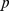 and the area 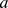 spanned by linguistic groups follow the allometric relation 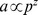, with an exponent 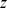 varying accross different world regions. The empirical evidence presented leads to the hypothesis that the distributions of 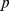 and 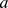, and their mutual dependence, rely on demographic dynamics and on the result of conflicts over territory due to group growth. To substantiate this point, we introduce a two-variable stochastic multiplicative model whose analytical solution recovers the empirical observations. Applied to different world regions, the model reveals that the retreat in home range is sublinear with respect to the decrease in population size, and that the population-area exponent 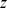 grows with the typical strength of conflicts. While the shape of the population size and area distributions, and their allometric relation, seem unavoidable outcomes of demography and inter-group contact, the precise value of 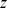 could give insight on the cultural organization of those human groups in the last thousand years.

## Introduction

Despite the extraordinary sociocultural diversity of human communities [1], a number of statistical features characterizing human populations follow remarkably simple patterns. Often, those patterns arise from basic mechanisms with a relatively small contribution of cultural traits. As examples we find the distribution of city sizes across the world –grounded in the fluctuating growth and decline of their populations [2]– or the use of space in hunter-gatherer groups –which relies on metabolic and environmental constraints and group dynamics [3]. A macroecological approach to social organization focusing on the exchange of energy and resources among individuals and societies, and with their environment, is a promising avenue toward a quantitative understanding of human space use, of population structure, and of cultural and linguistic diversity patterns [4].

One of the most basic traits that define a group of human beings is sharing a common language: the evolution of languages can be closely mapped onto the evolution of cultural groups despite the fact that, at present, the number of the latter is almost certainly larger than that of languages [1]. Actually, studies of linguistic phylogenies can be as informative as studies on gene evolution [5], [6], and linguistic taxonomy presents scaling properties [7] analogous to those of biological taxonomy [8], [9]. The similarities between biological and linguistic patterns go beyond a mere analogy [10]–[12]. As with biological species richness [13], linguistic diversity attains its maximum value close to the equator [14] and decreases with latitude. Ecological processes, historical factors, and home ranges for biodiversity [15], as well as environmental variables and political complexity for ethnolinguistic groups [16], have been put forward as the main drivers behind tropical richness. Still, there is no agreement on the origin of the latitudinal gradient in species richness [17], probably indicating that, in this case, several different mechanisms contribute in similar amounts.

A quantity related to linguistic diversity is the average area (or home range) covered by a human group. Data on the geographic ranges of languages [18] show that the area covered by a group increases with latitude [14] and thus follows Rapoport’s rule, which describes this same phenomenon for home ranges of biological species [19]. Beyond environmental variables, the home range of ethnnolinguistic groups has been found to depend on their subsistence strategy and on their political complexity [16]. In the case of hunter-gatherers, the home range as a function of group size has been taken as an indicator of its environmental needs and of its social behavior [3].

Though the home range is an important feature characterizing biological species or human groups, there is a quantity that seems to be more directly affected by environmental characteristics and cultural practices: the size of the group, measured by the number of members. An analysis of the correlation between the sizes 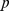 of linguistic groups and the areas 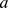 over which they spread reveals that both variables are significantly correlated and fulfill an allometric relationship 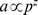, with a variable exponent 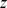. The distribution of abundance and range of biological species fullfils an analogous relationship, though there is no agreement on the processes originating it [20], [21]. In this paper, we present evidence on the universality of the relation between population and area for human groups and suggest, on the basis of a well-motivated mathematical model, that this relationship, as well as the functional form of the distribution of areas, stems from demographic processes and the interaction between neighboring groups.

### Distribution of Sizes of Linguistic Groups

**Figure 1:**
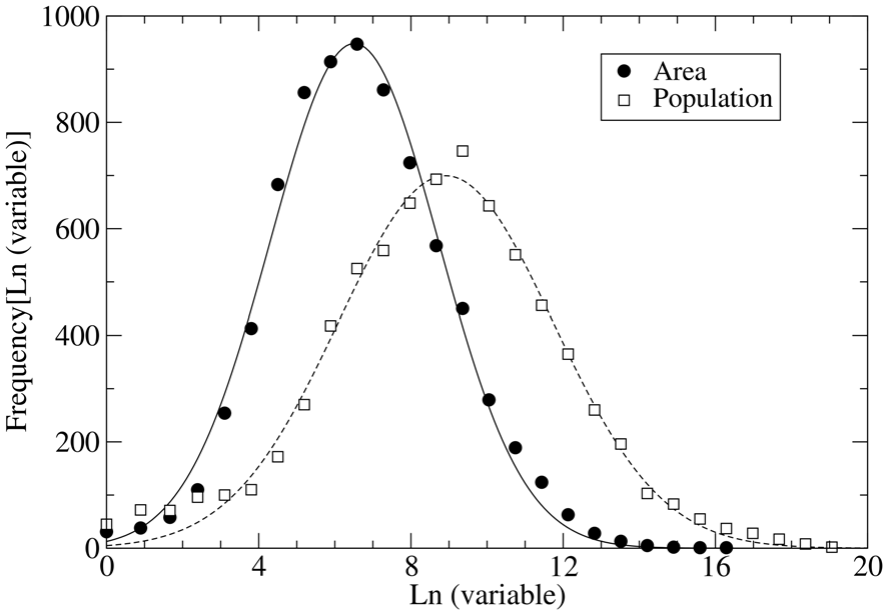
Normalized frequency distribution of language sizes and language areas (home ranges) over the world. When a language is spoken in two or more disconnected domains, the total area (sum of all disconnected polygons) is represented. The data set includes all living languages plus about 700 recently extinct languages listed in the Ethnologue [22]. Curves stand for Gaussian fittings on the logarithmic variables. Both fittings are statistically highly significant, yielding correlation coefficients 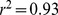 for the distribution of population sizes and 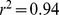 for the distribution of areas.

According to linguists’ classifications, there are about 6900 languages currently spoken all over the world [22]. Among them, 516 are classified as nearly extinct (with less than 100 speakers each), while the ten most abundant languages are spoken by a total of 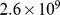 people. The uneven distribution of language sizes [23], [24], measured as the number of speakers per language (see Fig. 1), is well described by a log-normal frequency distribution [11]. Only rare languages deviate significantly from the lognormal shape and are more frequent than expected. Early analyses of the distribution of the number of individuals in a given biological species pointed out to a log-normal distribution as well [25], though, subsequently, other distributions for species abundance have been also observed in different ecosystems [26].

It is estimated that the population of the world in year 1000 was 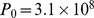 [27]. This number has grown at least eighteen-fold in ten centuries to reach the 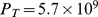 people accounted for by the Ethnologue in year 2000 [22]. The log-normal distribution of language sizes can be explained by means of a model that considers the stochastic multiplicative growth of independent populations in the last thousand years as the main mechanism behind the evolution in size of linguistic groups [28]. The only parameters of the model are those retrieved by assuming an exponential growth of the world population from 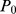 to 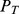, and the current distribution of language sizes, yielding for the annual growth rate a mean value 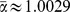 and a mean square dispersion 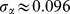 [28]. Simple models for the distribution of biological species abundance also rely on demographic dynamics, though they typically keep the total population bounded [26], [29].

## Results

### Home Range Distribution

**Figure 2:**
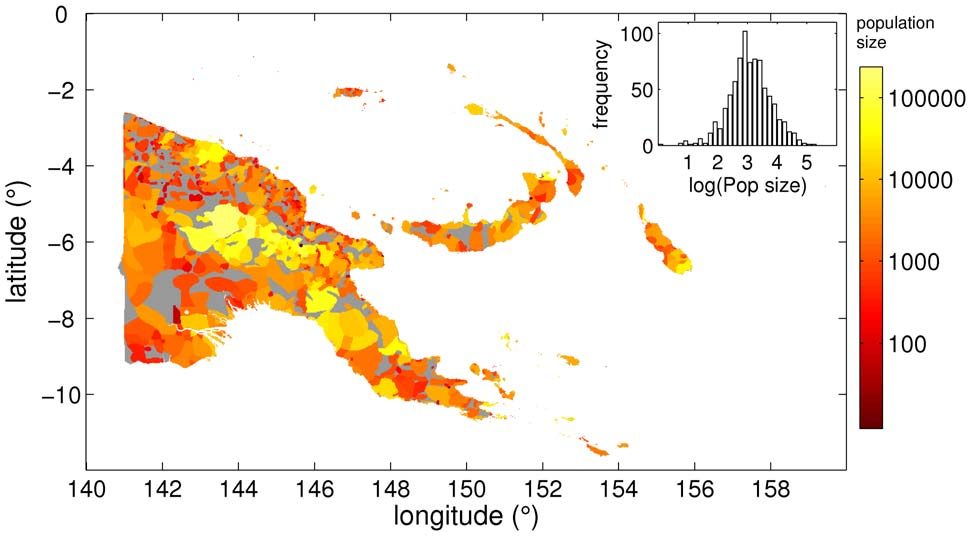
Linguistic diversity in Papua New Guinea and surrounding islands. Each polygon corresponds to a domain where a single language is spoken. The matrix where polygons are embedded (see Materials and Methods) has been mapped to an indexed image using 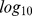 transformation of the population sizes for each language, as catalogued by SIL in the Ethnologue DB ver.15. The index values are colored according to the scale in the right, which indicates the absolute size of the group speaking each language. Unhabited landmass of Papua New Guinea is colored grey. The inset contains the population size histogram, with six bins per decade from 1 to 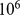.

Data on the geographical distribution of languages have been obtained from the fifteenth edition of the Ethnologue [22]. In order to avoid boundary effects due to small islands and coasts, only languages whose centroids are located more than 5 kilometers inland and belong to the 100 largest terrestrial landmasses have been considered (see Materials and Methods). An example of how territory is fragmented into different linguistic groups is shown in Fig. 2, which also illustrates the quality of the data analyzed.

The areas covered by linguistic groups also follow a broad distribution compatible with a log-normal function (Fig. 1). This is a remarkable observation considering the plethora of different mechanisms that underlie the spread of languages and the existence of patterns such as described by Rapoport’s rule [19]. In analogy with the distribution of language sizes, one may hypothesize that the variation in the home range of a group should be described as well by a multiplicative process. However, the justification of such a mechanism is not as natural as it appears for the size of linguistic groups, where population growth and decline are chiefly driven by the multiplicative stochastic effect of birth and death events. Should demographic dynamics be also behind the observed distribution of language areas, population sizes and home ranges should be significantly correlated.

### Relationship between Linguistic Group Size and Area

**Figure 3:**
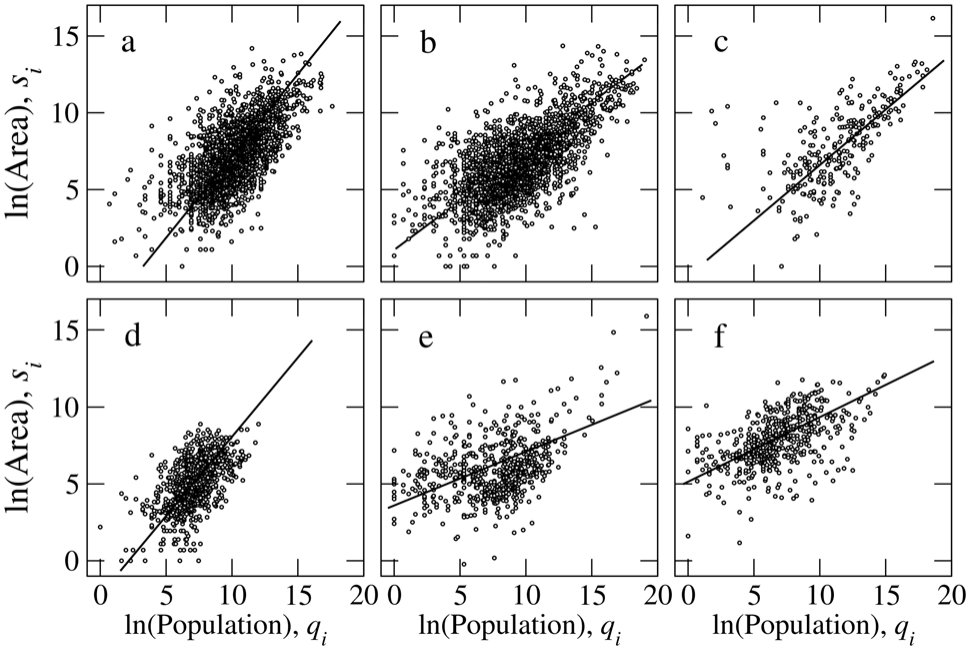
Scatter plot of the logarithm of the area (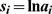) and the logarithm of the population size (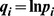) for all languages. Six different representative regions are plotted: (a) Africa (2314 languages), (b) Asia (2333 languages), (c) Europe (260 languages), (d) Papua New Guinea (813 languages), (e) North America (585 languages), and (f) South America (517 languages). North and South American languages have been separated according to whether their centroids laid to the north or to the south of the 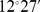
N parallel, which stands for the northernmost point of South America. The corresponding values of 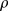 and 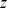 are compiled in Table 1. The value of 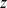 for each case coincides with the slope of the line drawn in each panel. Axes labels and sizes are the same in all cases.

Following the observation that the size 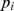 of the population speaking language 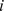 and the area 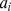 it covers are log-normally distributed –and likely correlated– we analyzed the dependence between both variables, as shown in Fig. 3. Six different representative regions are shown: Africa, Asia, Europe, Papua New Guinea, and North and South America. In all cases, the correlation between the two variables is high, though it is also affected by a substantial dispersion.

The log-normal distributions of language sizes and areas imply that the transformed variables 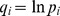 and 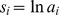 are well fitted by Gaussian distributions. Their joint distribution can be approximated by a bivariate normal distribution (BND, see Materials and Methods). The two main quantities characterizing this joint distribution are the exponent 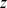, which yields the slope of the major ellipse axis of the scatter plot (and is represented as a straight line in all plots of Fig. 3), and the coefficient 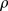, which quantifies the degree of correlation between the two variables: the larger 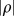, the narrower the ellipse and the more correlated 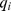 and 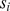 are.

A summary of the quantities characterizing each of the regions analyzed is shown in Table 1. The first four columns contain the averages 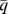 and 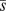, the slope 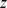, and the area-population correlation 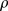 for human languages in each of the six regions mentioned, for the whole world, and for modern populations of hunter-gatherers (see below). As can be seen, the variation in the exponent 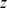 characterizing the allometric relationship is broad, while the correlation between population sizes and areas is similar in all cases, as shown by the narrow range spanned by 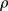.

**Table 1:** Parameters characterizing the distribution of population size and area covered by human languages in several representative world regions.

	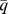	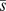	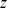	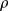	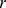	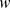
Africa	9.63	7.13	0.94 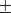 0.02	0.64 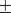 0.01	0.75 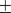 0.02	2.30 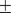 0.04
North America	7.61	5.97	0.35 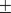 0.03	0.40 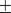 0.03	–	–
South America	6.76	7.82	0.42 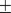 0.03	0.53 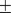 0.03	0.11 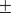 0.01	1.59 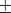 0.10
Asia	9.07	6.66	0.64 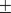 0.01	0.66 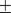 0.01	0.67 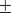 0.01	1.73 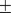 0.03
Europe	10.31	7.85	0.71 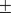 0.05	0.64 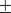 0.04	0.63 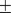 0.04	1.92 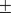 0.12
Papua N G	7.10	5.00	1.03 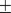 0.05	0.56 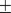 0.02	0.34 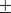 0.02	2.61 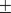 0.16
**World**	**8.91**	**6.51**	**0.57 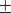 0.01**	**0.52 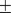 0.01**	**0.100 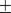 0.003**	**1.92 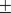 0.04**
Hunter-gatherers	6.73	4.57	2.02 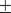 0.10	0.48 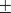 0.03	0.020 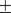 0.002	3.84 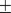 0.18

Columns represent the following data: 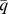 and 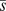 are the (logarithmic) averages of population size and area; 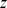 corresponds to the slope of the major ellipse axis relating 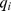 and 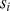, and 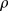 measures their degree of correlation. Errors in both variables are shown. The values of 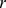 and 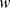 correspond to model parameters yielding the measured values of 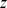 and 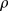 within the estimated interval.

### Model

The occurrence of a BND for the distribution of the logarithms of population sizes and areas for human languages plausibly suggests that their joint evolution can be accounted for by a stochastic multiplicative process correlating the two variables. Specifically, the demographic pressure associated with population growth of a given human group should promote its geographical expansion. This tendency, however, will generally clash with similar trends in neighboring populations and a conflict may ensue due to the limited area available. On the other hand, a decrease in population numbers leads to a contraction of the group, with the consequent decrease in the occupied area.

Let us assume that the (logarithmic) number of individuals in a human group, characterized by their common language, obeys the recurrence equation.
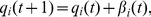
(1)where the index 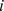 identifies the group, and 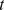 is measured in years. The additive process (1) is the direct translation of the multiplicative process on the original variable 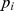. At each step, the growth rate 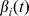 is randomly drawn from a uniform distribution 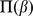 in the interval 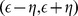 (see Materials and Methods). The half-width 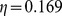 and the mean value 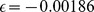 are taken from the average evolution of linguistic groups in the last thousand years [28].

Similarly, the evolution of the corresponding (logarithmic) area is assumed to obey.
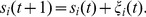
(2)


The key ingredient of the model is how the dynamics of 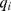 and 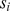 are correlated or, in other words, how the corresponding growth rates depend on each other. We assume that 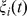 depends on the growth rate of the population size, 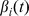, according to the following rules:

1. If 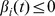, 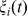 is drawn at random from a uniform distribution in the interval 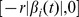. This represents a shrinking of the area when the population decreases.

2. If 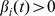, the group 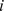 enters into conflict with other groups. To represent a typical conflict event, a second group 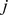 is randomly chosen and the growth rates of 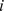 and 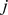 in the last year are compared:

(a) If 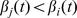, 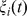 is drawn from the interval 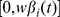. Since group 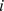 presents the largest growth rate, it prevails and its area grows.

(b) If 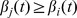, 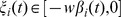. Group 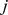 prevails, and 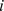
’s area shrinks.

According to these rules, the stochastic evolution of 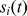, which becomes correlated to that of 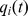, turns out to be fully determined by the two parameters 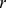 and 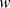, respectively quantifying the spontaneous retreat and the outcome of conflicts, and by the distribution 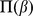. The exponent 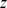 and the correlation 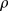, which quantify the statistical interdependence of language sizes and areas, can be written as functions of the model parameters in analytical form (see Materials and Methods). For each choice of 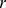 and 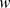, the model yields a single pair 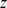, 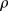.

In the present model, the number of human groups –and, therefore, of different languages– is preserved along the evolution, so that such events as language birth, fragmentation and death are not explicitly taken into account. Language extinction, which arguably was the most important among these processes in the last thousand years [11], [22], can nevertheless be introduced by eliminating those groups whose population falls below a prescribed level, for instance, of one individual. However, we have verified by means of numerical simulations that this additional ingredient has no significant effect on the resulting values of 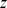 and 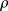. On the other hand, the model does not exclude that a language evolves along time, as long as it preserves its identity through association with a given human population.

Note that the above rules impose no restriction on the growth of the geographical region occupied by each population. Thus, the total area covered by the system can increase indefinitely. This feature, which is manifestly unrealistic for recent stages in the evolution of human populations, can be corrected by assuming any prescribed time dependence for the total area –for instance, that it remains constant or that it grows monotonically approaching a limit value– and, accordingly, rescaling individual areas at each evolution step. This rescaling does not affect the correlation between areas and populations and, therefore, the values of 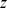 and 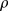 are not modified.

### Expected Values of 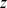 and 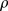


**Figure 4:**
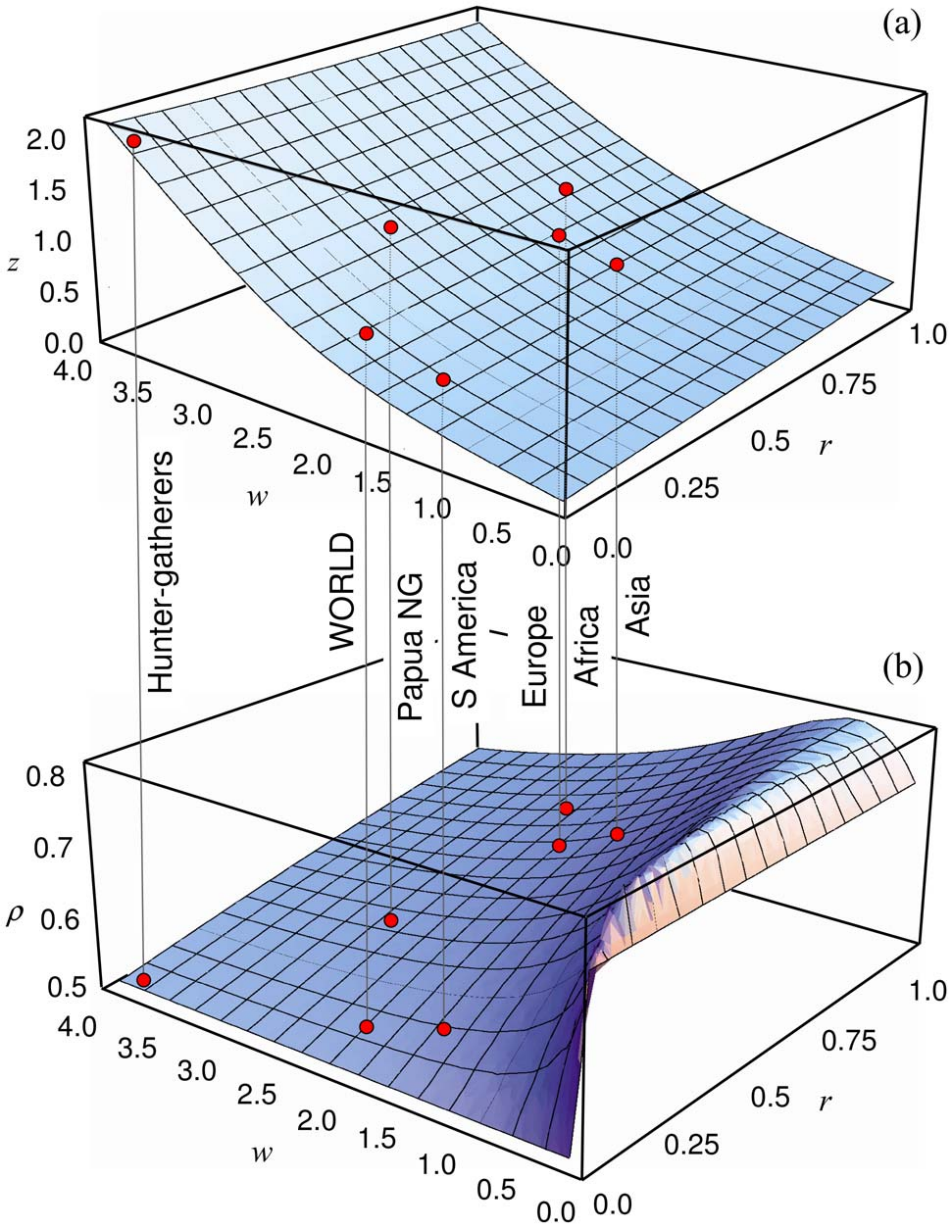
Model results. (a) Exponent 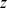 and (b) correlation 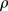 yielded by the model as a function of the parameters respectively characterizing spontaneous retreat and conflict outcome, 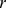 and 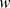. The population-area relationship is recovered with different parameters for all world regions, except for North America (not shown in the plots). Data for the world considers all points represented in Fig. 3. Though 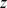 and 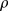 are, in principle independent variables (in terms of an arbitrary BND), the model establishes a quantitative relationship between them. The allowed values of 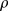 when 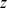 remains fixed can be read from this figure, and *vice versa*. As an example, note that, if 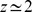, 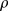 is to be found in the range 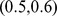, approximately.

The two surfaces represented in Fig. 4 correspond to the values of 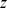 (Fig. 4(a)) and 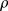 (Fig. 4(b)) obtained by numerically solving the exact equations for the two variables in the intervals 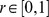 and 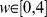. Except for North America, whose area-population correlation is sensibly lower than for other regions, the model can quantitatively recover all empirical values for the groups included in Table 1.

## Discussion

The size of linguistic groups and the area they cover are well fitted by log-normal probability distributions. The correlation between those two quantities supports that the demographic process defined by natural population growth is the main mechanism behind those distributions. The correlation between group size and area, described by our index 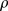, is close to 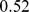 (world average) for almost all cases analyzed. A multiplicative model relating the evolution of group sizes and areas is able to explain and quantitatively recover the observed patterns. In our analysis of the model, the exact equations for 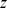 and 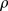 were actually solved in a range of values of parameters 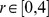 and 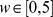 significantly broader than those shown in Fig. 4. Nevertheless, those pairs recovering empirical data fulfill 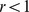 and 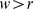 (see Table 1 and Fig. 4). Values of 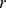 smaller than one imply that the decrease in area when a population shrinks is sublinear with respect to the decrease in population size. Though demographic change may be a primary cause behind areal variations, other causes such as the use of primary resources or the cultural value of the territory likely act against reducing the home range. On the other hand, 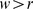 indicates that the loss or gain in area experienced by two conflicting populations is larger than the change they would undergo when no conflict arises. This is also a plausible result of group clash in comparison to spontaneous retreat.

Additional support to the plausibility of the dynamical rules implemented in our model comes from independent data of 339 present-day traditional hunter-gatherer groups [18]. Despite the cultural control of natality they practice, the distributions of sizes and home ranges of those traditional societies are remarkably well fit by log-normal distributions. A covariance analysis of the relationship between the home range and the group size yields 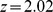 and 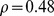.

The special case of native North American languages, whose value of 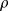 cannot be recovered by the model, can be however qualitatively understood. Most of the linguistic territories reported by the Ethnologue database in the United States and in Canada correspond to very small areas, assimilable to reduced aboriginal communities and reservations (compare 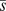 in North America to other continents). In these artificially created ethnic domains, it is expected that the (assigned) areas are much less correlated to the population size than in groups whose spatial distribution is governed by demographic dynamics. This explains the small value of 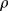. Artificial territorial allowance may also explain the relatively small slope 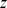, because of the limited possibilities of expansion. The population in such communities, in any case, is systematically depleted by emigration toward and assimilation by urban settlements of European origin. Consistently, other analyses of the mechanisms underlying the spread of ethnolinguistic groups have explicitly removed languages in the Americas and in Australia (which show restricted ranges) due to their colonial history and population replacement [16].

Though we cannot interpret the relation between the model parameters 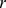 and 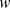, and the empirical quantities 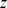 and 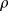 in a straightforward way, there is an eye-catching correlation between the values of the slope 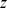 and those of 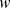: the stronger the effect of a conflict, the larger the population-area exponent 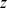 (see Fig. 4(a)). Current data support that the relative number of casualties in violent conflicts has declined with the advent of modern states: *pre-state societies were far more violent than our own. 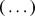 In tribal violence, the clashes are more frequent 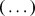 and rates of death per battle are higher* [30]. In zones of anarchy, violence is also higher than in regions ruled by stable governments [31]. It seems reasonable that the fraction of population that perishes in violent conflicts correlates positively to changes in owned territory. If this is so, larger values of 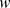 should better describe those regions which, along the historical period relevant to the demographic processes discussed here, were socially less organized, and *vice versa*.

Though, admittedly, our model does not consider all the processes affecting the population-area relationship in human groups, the conclusions drawn from its solution are robust under changes such as group extinction (when the population falls below a single individual) or under quantitative changes in 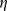 and 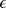. We have used average values for 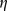 and  (which come from data corresponding to world population) and have applied them to all regions. Those two quantities and other relevant parameters as 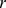 and 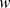 almost certainly vary in time, while we have considered them to be constant. Analyses aimed at predicting the fate of specific (small) regions should take into account region- and time-dependent parameters, and explicitly model the spatial allocation of population density. Also, since the location of different populations with respect to each other was disregarded, we have considered that interaction between any two groups is equally probable, while in reality conflict is much more likely to arise between neighboring groups.

Certainly, environmental variables, subsistence strategies, cultural behaviour or political complexity play a role in determining the spread of linguistic groups. These facts do not contradict our main hypothesis, namely, that demographic dynamics and conflict explain the allometric relationship between group size and area, and their correlation. Specific environmental and cultural variables may be instrumental in determining which particular groups grow and which others shrink, and very likely do condition the outcome of conflicts. In this sense, their predictive power in particular cases is far beyond that of our statistical model. Still, we maintain that simple processes as the ones here implemented might be the main drivers to ascertain how human groups are distributed in space, and to what extent their statistical patterns resemble those observed in non-cultural species.

## Materials and Methods

### Data Origin

Data on the size of linguistic groups and their geographical properties have been obtained from the World Language Mapping System (WLMS 3.2) of the Ethnologue, version 15 by The Global Mapping Inc. (http://www.worldgeodatasets.com/). Permission to publish the processed data was received. Considered regions correspond to polygons with centroids located more than 5 kilometers inland of the shoreline. The shoreline is defined in the Global Self-consistent, Hierarchical, High-resolution Shoreline Database (GSHHS) by The National Oceanic and Atmospheric Administration (NOAA, http://www.ngdc.noaa.gov/mgg/shorelines/gshhs.html). Polygons were embedded in a matrix at a resolution of 48 pixels per degree using the ‘poly2mask’ function in MATLAB. Areas were calculated using the ‘areaint’ function of the Mapping Toolbox in MATLAB version 7.

### Bivariate Normal Distribution

The joint distribution of two variables 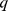 and 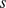 that independently follow Gaussian distributions can be approximated by the bivariate normal distribution (BND),
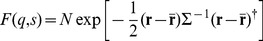
(3)with 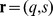, 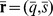, 
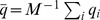
, 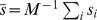, and 
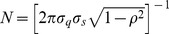
. 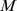 is the number of data points, and the index 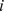 runs over these data. The covariance matrix 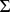 is given by
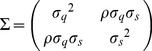
(4)with 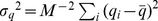, 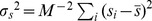, and 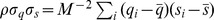. The eigenvectors of 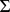 can be written as 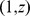 and 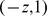, where 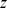 corresponds to the slope of the major ellipse axis of the scatter plot. The value of 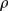 quantifies the degree of correlation between the two variables: the larger 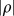, the narrower the ellipse.

### Analytical Solution of the Model

The marginal distribution for the population growth rate, 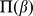, which is uniform over the interval 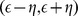, can be written as
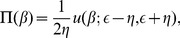
(5)where 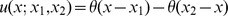 and 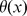 is the Heaviside step function. Then, the joint distribution function for the growth rates of populations and areas is



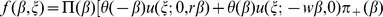


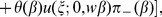
(6)with 
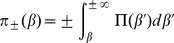
.

By virtue of the central limit theorem, for sufficiently long times (i.e. when the contribution of the initial conditions can be neglected), the mean values and the (co)variances of 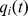 and 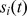 will be given by 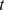 multiplied by the respective quantities for 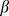 and 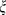 over their joint distribution 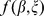. Consequently, the correlation 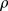 and the slope 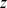 of the (logarithmic) populations and areas will be the same as those corresponding to 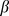 and 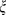. In other words, in the long-time limit, 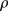 and 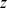 can be expressed in terms of 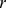, 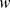, and the parameters 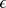 and 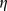 in 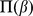. We recall that, as stated above, the latter can be fixed from real data on the evolution of human populations. The expressions for 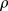 and 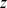 in terms of 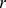 and 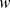 are algebraically very involved but, nonetheless, can be found analytically and dealt with by standard computational means.

### Estimation of Errors

Errors in 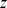 and 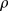, as reported in Table 1, were obtained by standard bootstrapping techniques. Given the values of 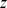 and 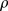 obtained from the best fit of the bivariate normal distribution for each world region, we extracted a set of data points at random from the predicted distribution. For the random set, whose number of data equaled that of the corresponding world region, the values of 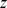 and 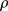 were recalculated. The assigned errors were the dispersions in the distributions of 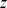 and 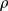 obtained from 1000 surrogate data sets. The errors in the model parameters 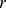 and 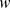 have been obtained from the analytical relationship provided by the analytical solution of the model.
